# Self-Reported Musculoskeletal Disorders of the Distal Upper Extremities and the Neck in German Veterinarians: A Cross-Sectional Study

**DOI:** 10.1371/journal.pone.0089362

**Published:** 2014-02-19

**Authors:** Agnessa Kozak, Grita Schedlbauer, Claudia Peters, Albert Nienhaus

**Affiliations:** 1 Institute for Health Services Research in Dermatology and Nursing, University Medical Center Hamburg-Eppendorf, Hamburg, Germany; 2 Department of Occupational Health Research, Institution for Statutory Accident Insurance and Prevention in Healthcare and Welfare, Hamburg, Germany; Georgia Regents University, United States of America

## Abstract

**Background:**

Veterinary work is a physically demanding profession and entails the risk of injuries and diseases of the musculoskeletal system, particularly in the upper body. The prevalence of musculoskeletal disorders (MSD), the consequences and work-related accidents in German veterinarians were investigated. Work-related and individual factors associated with MSD of upper extremities and the neck were analyzed.

**Methods:**

In 2011, a self-reporting Standardized Nordic Questionnaire was mailed to registered veterinarians in seven federal medical associations in Germany. A total of 3174 (38.4%) veterinarians responded. Logistic regression analysis was used to determine the association between risk factors and MSD-related impairment of daily activities.

**Results:**

MSD in the neck (66.6%) and shoulder (60.5%) were more prevalent than in the hand (34.5%) or elbow (24.5%). Normal activities were affected in 28.7% (neck), 29.5% (shoulder), 19.4% (hand) and 14% (elbow) of the respondents. MSD in the upper body occurred significantly more often in large animal practitioners. Accidents that resulted in MSD were most frequently reported in the hand/wrist (14.3%) or in the shoulder (10.8%). The majority of all accidents in the distal upper extremities were caused by animals than by other factors (19% vs. 9.2%). For each area of the body, a specific set of individual and work-related factors contributed significantly to severe MSD: Older age, gender, previous injuries, BMI, practice type, veterinary procedures such as dentistry, rectal procedures and obstetric procedures as well as high demands and personal burnout.

**Conclusion:**

From the perspective of occupational health and safety, it seems to be necessary to improve accident prevention and to optimize the ergonomics of specific tasks. Our data suggest the need for target group-specific preventive measures that also focus on the psychological factors at work.

## Introduction

Recent studies on professional veterinarians have demonstrated that veterinary work is physically demanding and poses an elevated risk of significant injuries [1]–[4]. A number of physical and psychological risk factors at work, particular in veterinary professions, have been linked to musculoskeletal disorders (MSD): static or awkward postures, repetitive or forceful tasks, animal related injuries, pressure of time, work stress, career structure or after hours duties [5]–[8]. Equine and bovine practitioners regularly undertake repetitive tasks, such as rectal palpation or obstetric procedures, which require lifting or exerting an upward force and/or resisting animals’ unpredictable movements [4]. Some practitioners work with one or both arms above shoulder level for over one hour daily [7]. These postures and movements may be risk factors for the development of MSD in the upper extremities in veterinarians [6], [9]. Ailsby, for instance, assumed that an occupational neck- shoulder- and arm-syndrome is significantly associated with continuous or repeated strain from repetitive and forceful motions during veterinary work (e.g. procedures such as rectal examinations or calving) [10]. Such microtrauma or minute injuries from repeatedly overusing a specific part of the body result in conditions called “Repetitive Strain Injuries” or “Cumulative Trauma Disorders” (CTD). In the literature, those conditions are summarized under the higher level term of “Work-Related Musculoskeletal Disorders of Upper Extremities” (WRMSDs-UE), which are inflammatory and degenerative disorders responsible for pain and functional impairment in tendons, muscles, joints, nerves or blood vessels [11].

Work-related accidents constitute a further health risk factor. According to the insurance data from the Statutory Accident Insurance in the Health and Welfare Services in Germany (BGW- Berufsgenossenschaft für Gesundheitsdienst und Wohlfahrtspflege) for the 5-year period (1998–2002) of all reported claims, work-related accidents accounted for 87.7% of claims in veterinary practice. Animals (66%) were the main cause of these accidents [12]. Numerous studies have shown that veterinarians are at high risk of significant acute traumatic injury (ATI) from animal contacts - predominantly in the upper extremities [2], [13]–[15]. Large animal practitioners are most likely to suffer severe injuries [8], [16]. In particular, palpation is one of the five most common causes of injuries in veterinary practitioners [13]. The risk of job-related injuries in veterinarians is higher than for other professions in the healthcare sector [12].

Therefore, working characteristics may contribute to the prevalence of MSD symptoms in veterinarians. They may suffer from work-related physical impairment or disability in functional tasks and/or the chronic or acute musculoskeletal disease. Veterinarians are more likely to report chronic work-related musculoskeletal problems if they perform clinical work [8]. According to the registration data from the German Federal Veterinary Council, 49% of the veterinarians in Germany work in clinical practice and perform tasks and procedures which can lead to MSD [17]. However, there are no available data on the prevalence of MSD in German veterinarians.

The purpose of the present study was, therefore, to examine the self-reported prevalence for MSD, the resulting physical limitations, and the frequency of injuries in the distal upper extremities and the neck. We also investigated the relationship between demographic, occupation-related risk factors (e.g. practice type, work task or previous injury) and MSD-related impairment of daily activities (severe MSD) in the relevant body regions.

## Materials and Methods

### Subjects

In 2011 we conducted a survey of registered veterinarians in seven federal states in Germany (medical associations in Baden-Württemberg, Bavaria, Berlin, Brandenburg, Lower Saxony, Schleswig-Holstein, and Westphalia-Lippe). According to the registration data from the German Federal Veterinary Council, there were no statistically significant differences between responders and non-responders with respect to gender and age distribution [17]. None of the seven medical associations of the federal states in the study was overrepresented. Thus, the sample can be considered as representative.

### Measurement

We measured the presence and severity of MSD during the preceding 12 months with the Standardized Nordic Questionnaire [18]. This instrument has been applied to various occupational groups to evaluate musculoskeletal problems. An anatomical sketch of labeled body regions allowed the respondents to clearly identify body areas affected by MSD. A dichotomous yes/no answer indicated whether the participants had suffered any symptoms in the queried body area during the preceding 12 months. To assess the severity, the following question was asked: *“What is the total length of time that [neck] trouble has prevented you from doing your normal work (at home or away from home) during the last 12-months?”.* Possible replies to this question were: (1) *“the discomfort was not too severe”;* (2) *”1 to 7 days”;* (3) *”8 to 30 days” or* (4) *”more than 30 days”.* We aimed to distinguish participants who felt a certain discomfort but did not experience longer lasting restrictions in work and daily life from those who were restricted for at least one week in the past 12 months. Participants who replied with “*the discomfort was not too severe”* were allocated to the non-affected group. For each queried body area, we also asked the participants whether these symptoms were caused by a work-related injury during their professional life. Work-related injuries were differentiated into animal-related and other-related injuries during working hours (e.g. falls, motor vehicle accidents). The participants were asked to provide their demographic data (age and gender), anthropometric measures (height and weight), dominant hand, current job status (full-time or part-time), the type of practice (small, mixed or large animal), length of work experience, number of hours worked per week and whether they were involved in sports activities or not. We calculated the body mass index (BMI) and categorized it into normal weight (BMI<25), overweight (25–30), and obese (>30).

The veterinarians were asked to estimate the number (0; <600; 600–2400; 2400–6000; 6000–12,000; >12,000) of examinations and procedures they performed annually. The detailed list of veterinary procedures was adopted from Scuffham et al. and translated into German [4]. The following procedures were queried: (1) obstetric procedures, (2) rectal palpations, (3) inseminations, (4) vaginal examinations, (5) animal handling/lifting, (6) blood sampling, (7) vaccinations, (8) dehorning/velveting, (9) foot trimming, (10) lameness examinations, (11) necropsies, (12) ultrasonography, (13) radiography, (14) endoscopies, (15) dental procedures, (16) surgery lasting <1 hour, and (17) surgery lasting >1 hour. For the analysis, we summarized the number of procedures into three categories (0 or not specified; <600; ≥600).

Quantitative job demands were measured by a five-point Likert scale from the Copenhagen Psychosocial Questionnaire (COPSOQ) [19]. This scale measures the amount of work that has to be done in a particular time (e.g. intensive and extensive job demands). The frequency of typical load factors at work were measured with four questions (1. *“Do you have enough time for your work tasks?”;* 2. *“Do you have to do overtime?”;* 3. *“Is your work unevenly distributed so it piles up?”;* 4. *“Do you have to work very fast?”).* The Cronbach’s Alpha value was acceptable (α = .65). To capture the psychological condition of the veterinarians, the ‘personal burnout’ subscale from the Copenhagen Burnout Inventory (CBI) was applied. This scale contains six items on general symptoms of exhaustion and is defined as “the degree of physical and psychological fatigue and exhaustion experienced by the person”. This five-point Likert scale (α = .87) shows good internal consistency [20]. Both scales were transformed into a theoretical range, extending from 0 (never/almost never) to 100 (always) points. This transformation is a standardized procedure and conforms to the German validation study. If at least half of the single items had valid answers, scale scores were computed as the average of the values [21]. For the logistic regression models, we summarized the original scales into tertiles and defined them as low, medium or high. The final questionnaire was pre-tested on ten veterinarians to remove inconsistencies, detect unclear wording and to complement missing aspects.

### Ethics Statement

Each questionnaire included an informative letter which clarifies the free participation and anonymity of this study. We did not ask for the written consent of the participants. The voluntary participation was deemed as informed consent. The study protocol was approved by the Hamburg Medical Council Ethics Commission (# PV3839).

### Statistical Analysis

Descriptive statistics were used to describe the study sample and to estimate the prevalence of musculoskeletal disorders (MSD), the disorder severity (activities affected) and accident prevalence in the upper extremities and neck. Differences in MSD prevalence were examined using Pearson’s chi-square test for categorical variables. For collinearity analysis, we scanned the Spearman correlation matrix to avoid multicollinearity or redundancy between independent variables (e.g. job tasks). Spearman’s correlation coefficients of *ρ*≥0.6 were considered as problematic, as they introduce a substantial bias into the estimation of the logistic regression models [22]. Thus, the variable ‘job experience’ was removed from the analysis, as this strongly correlated with age (*ρ* = 0.8). We found that rectal palpations were frequently mentioned as a risk factor for CTD or ATI in the upper extremities [3], [6], [13]. As the predictor variables foot trimming, dehorning/velveting, vaginal examinations, and inseminations showed significant moderate correlations with rectal palpations (*ρ*≥0.6), we decided to remove these from further analysis. Univariate logistic regressions were calculated to identify associations between severe MSD in the previous 12 months and individual, work-related and psychosocial factors in the respective body parts. As we performed a number of tests, we set the alpha level at p<0.01, in order to lower the probability of type I errors. Predictor variables which significantly affected the rate of MSD severity were selected for multivariate modeling. However, the demographic variables age and gender were included in each tested model, whether or not they had a significant influence in the univariate analysis. Backward stepwise multivariate logistic regression analyses were performed to develop a final explanatory model for each body part. The likelihood ratio statistic was used for variable entry (p<0.05) and removal (p<0.1). Analyses were performed with SPSS Version 17.0.

## Results

### Study Population

A total of 3174 veterinarians responded to the self-administered questionnaire (response rate 38.4%). Complete information was provided by 3051 subjects (96% of the responses). Table 1 describes the study population. The mean age of the participants was 47.6 (±10) years, with an average of 18.0 (±10.2) years of job experience in veterinary practice. Of the 3051 participants in the survey, the majority (97.1%) worked in a clinical practice and 82% were self employed. Most study participants were small animal practitioners (48.6%). The proportion of women working in small animal practices was significantly higher (75.2%) than in mixed (38.9%) and large (31.8%) animal practices (p<0.001) and vice versa (25% of men worked in small, 61% in mixed and 68% in large animal practices). Thus, there was a gender difference in the tasks performed in veterinary practice. Male practitioners significantly more often performed tasks related to a job profile in mixed and large animal practices (e.g. rectal palpations or obstetric procedures). Table 2 shows relevant veterinary tasks which significantly correlated with practice type (Spearman’s correlation of at least *ρ* = 0.2). The tasks are ranked according to their relevance (high to low correlation). Large and mixed animal practitioners more often performed foot trimming, rectal palpations, inseminations, dehorning/velveting, vaginal examinations and obstetric procedures. Small animal practitioners, however, more often performed x-rays, handling and lifting, dental procedures and surgeries.

**Table 1:** Characteristics of the study population.

Variables		N = 3051 (%)
Sex	Female	1657 (54.3)
	Male	1394 (45.7)
Age*	<30	105 (3.4)
	30–39	561 (18.4)
	40–49	1081 (35.4)
	>50	1304 (42.7)
	>50	1304 (42.7)
BMI	<25	1733 (56.8)
	25–30	1050 (34.4)
	>30	268 (8.8)
Job experience**	<10	693 (22.7)
	10–19	1003 (32.9)
	20–29	936 (30.7)
	>30	419 (13.7)
Practice type	Small	1483 (48.6)
	Mixed	614 (20.1)
	Large	954 (31.3)
Working h/week	>35 h	2471 (81.0)
	15–34 h	465 (15.2)
	<15 h	115 (3.8)
Work setting	Practice	2963 (97.1)
	Industry	57 (1.9)
	University/Administration	24 (0.8)
	Other	7 (0.2)

*Note.* *Age: mean 47.6 (SD±10) years; **Job experience: mean 18.0 (SD±10.2) years.

**Table 2:** The frequency of veterinary tasks performed annually, stratified by practice type.

Practice type	Small (%)			Mixed (%)			Large (%)		
Veterinary tasks	0	<600	600–2400	0	<600	600–2400	0	<600	600–2400
Foot trimming	77.8	6.4	1.2	12.8	31.9	25.3	9.5	61.7	73.5
Rectal palpation	81.8	60.7	6.3	9.8	19.7	29.6	8.4	19.6	64.1
Insemination	66.7	12.8	1.3	16.0	28.1	31.2	17.3	59.1	67.4
Dehorning/velveting	65.2	3.7	13.5	15.7	32.7	18.9	19.1	63.7	67.6
Vaginal examination	72.6	41.5	8.9	13.0	22.9	27.5	14.3	35.5	63.6
Radiography	22.4	55.2	69.7	20.0	22.3	15.1	57.6	22.5	15.1
Obstetric procedure	74.5	36.1	22.1	10.9	25.4	14.7	14.6	38.6	63.2
Handling/lifting	39.8	30.4	59.2	14.2	21.1	20.1	46.0	48.5	20.7
Dental procedure	22.4	54.7	57.7	11.8	22.9	18.6	65.8	22.4	23.7
Surgery lasting <1	40.7	44.9	67.2	22.7	19.8	20.2	36.6	35.4	12.6
Surgery lasting >1	37.2	50.2	68.4	18.7	20.8	17.2	44.1	29.0	14.4

*Note.* The differences between practice types were significant (p<0.05) for all procedures.

### Prevalence of MSD Symptoms

Of those affected, a quarter reported MSD trouble in one body site, 56% reported two to three body sites, and 8% experienced MSD in four queried body sites. The prevalence of MSD by body site is given in Table 3, stratified by gender and practice type. The prevalences of the MSD symptoms in the upper extremities in the preceding 12 months differed considerably; the highest prevalences were observed in the neck (66.6%) and shoulder region (60.5%). MSD in the hand (34.5%) and elbow (24.5%) were less frequently reported. Neck symptoms were more likely to be reported by female veterinarians (p<0.001). Male veterinarians, however, significantly more often reported symptoms in the elbow (p<0.001). A significantly higher proportion of large animal practitioners reported MSD in the distal upper extremities than did practitioners in mixed and small practices (p<0.001).

**Table 3:** Twelve month MSD prevalence and severe MSD (activities affected) of the upper body, stratified by gender and practice type.

Body region		Neck		Shoulder		Elbow		Hand/Wrist	
12-month prevalence		MSD experience	SevereMSD	MSD experience	SevereMSD	MSD experience	SevereMSD	MSD experience	SevereMSD
		n (%)	n (%)	n (%)	n (%)	n (%)	n (%)	n (%)	n (%)
**Female n = 1657**	**Total**	1240 (74.8)	527 (31.8)	995 (60.0)	466 (28.1)	336 (20.3)	198 (11.9)	592 (35.7)	350 (21.1)
	Small n = 1115	839 (75.2)	356 (31.9)	653 (58.6)	293 (26.3)	196 (17.6)	116 (10.4)	360 (32.3)	219 (19.6)
	Mixed n = 239	172 (72.0)	78 (32.6)	149 (62.3)	75 (31.4)	54 (22.6)	31 (13.0)	96 (40.2)	55 (23.0)
	Large n = 303	229 (75.6)	93 (30.7)	193 (63.7)	98 (32.3)	86 (28.4)	51 (16.8)	136 (44.9)	76 (25.1)
**Male n = 1394**	**Total**	793 (56.9)	348 (25.0)	851 (61.0)	433 (31.1)	411 (29.5)	230 (16.5)	460 (33.0)	242 (17.4)
	Small n = 368	211 (57.3)	82 (22.3)	183 (49.7)	79 (21.5)	77 (20.9)	30 (8.2)	84 (22.8)	37 (10.1)
	Mixed n = 375	217 (57.9)	96 (25.6)	236 (62.9)	114 (30.4)	112 (29.9)	67 (17.9)	123 (32.8)	69 (18.4)
	Large n = 651	365 (56.1)	170 (26.1)	432 (66.4)	240 (36.9)	222 (34.1)	133 (20.4)	253 (38.9)	136 (20.9)
**Total N = 3051**		**2033 (66.6)**	**875 (28.7)**	**1846 (60.5)**	**899 (29.5)**	**747 (24.5)**	**428 (14.0)**	**1052 (34.5)**	**592 (19.4)**

### Severe MSD (Activities Affected)

Correspondingly, the perceived physical disability in functional tasks (severe MSD) during the preceding 12 months was highest in the neck (28.7%) and shoulder (29.5%), and lowest in the hand/wrist (19.4%) and elbow (14%). Women showed higher MSD severity in the neck (p<0.001) and also in hand/wrist (p<0.01), while men reported significantly higher severe MSD in the elbow region (p<0.001). The proportion of severe MSD in the upper distal extremities increased significantly with the size of the treated animals (Table 3).

### Reported Accidents

Work-related accidents that resulted in MSD complaints were most frequently reported in the hand/wrist (14.3%) and shoulder (10.8%); the least reported injuries were in the neck (6.6%) and elbow region (5.5%). Except for the neck, more accidents in the upper extremities were caused by animals than by other factors (19% vs. 9.2%). Male practitioners were more likely to report injuries in the neck (11.4% vs. 6.5%), shoulders (23.4% vs. 10.1%), elbows (20.7% vs. 12.6%), and hand/wrist (36.5% vs. 30.1%) than female practitioners. Except for the elbow, the accident rate increases proportionally (p<0.05) with the age of the practitioners. Large and mixed animal practitioners were more likely to report accidents in the neck (14%,13% and 3.6%, respectively), shoulder (24.2%, 25% and 6.4%, respectively) and elbow (23.2%, 17.8% and 10.4%, respectively, p<0.001) than small animal practitioners. No significant differences were found for accidents in the hand/wrist (35.2%, 36.2% and 29.6%, respectively; no Table).

### Risk Factors for Severe MSD

Logistic regression analysis was used to identify factors influencing the risk of MSD causing restricted movements and restricted daily activities during the preceding 12 months. The analysis was performed separately for individual regions of the upper extremities; the results are shown in Tables 4, 5, 6, 7. As some predictor variables are the same for different body regions, we describe the results together.

**Table 4:** Multivariate analysis of severe MSD in the neck.

Variables		%	Crude OR (95%CI)	Adjusted OR^†^ (95%CI)
Age (years)	<30	21.9	1	1
	30–39	31.0	1.6 (0.9–2.6)	1.5 (0.9–2.6)
	40–49	29.0	1.5 (0.9–2.4)	1.6 (1.0–2.7)
	>50	27.9	1.4 (0.9–2.2)	2.0 (1.2–3.3)**
Accidents	No	35.3	1	1
	Yes	55.5	2.3 (1.7–3.1)	2.1 (1.6–2.9)**
Dental procedures	0 or n/s	26.8	1	1
	<600	28.2	1.1 (0.9–1.3)	1.0 (0.8–1.3)
	≥600	34.3	1.4 (1.1–1.9)	1.4 (1.0–1.9)*
Quantitative demands	Low	18.4	1	1
	Medium	25.7	1.5 (1.2–2.0)	1.3 (1.0–1.8)
	High	36.7	2.6 (1.9–3.4)	1.7 (1.2–2.4)**
Personal Burnout	Low	13.6	1	1
	Medium	31.6	2.9 (2.4–3.6)	2.1 (1.7–2.7)**
	High	50.8	6.6 (5.1–8.6)	4.3 (3.2–5.8)**

*Note*. *p<0.05; **p<0.01. ^†^Gender had no effect in the final model.

**Table 5:** Multivariate analysis of severe MSD in the shoulder.

Variables		%	Crude OR (95%CI)	Adjusted OR^†^ (95%CI)
Age (years)	<30	21.0	1	1
	30–39	23.9	1.2 (0.7–2.0)	1.3 (0.8–2.3)
	40–49	29.6	1.6 (1.0–2.6)	2.1 (1.2–3.6)**
	>50	32.4	1.8 (1.1–2.9)	2.1 (1.2–2.0)**
Accidents	No	42.1	1	1
	Yes	55.5	1.7 (1.3–2.2)	1.6 (1.2–2.0)**
Obstetrics	0 or n/s	22.3	1	1
	<600	32.7	1.7 (1.4–2.0)	1.4 (1.2–1.8)**
	≥600	42.1	2.5 (1.6–3.9)	2.1 (1.2–3.5)**
Radiography	0 or n/s	33.1	1	1
	<600	26.1	0.7 (0.6–0.8)	0.8 (0.6–1.0)*
	≥600	32.3	1.0 (0.8–1.2)	1.2 (0.9–1.4)
Quantitative demands	Low	16.8	1	1
	Medium	27.1	1.8 (1.4–2.5)	1.7 (1.2–2.4)**
	High	37.3	3.0 (2.2–4.0)	2.0 (1.4–2.9)**
Personal Burnout	Low	18.5	1	1
	Medium	31.5	2.0 (1.7–2.5)	2.0 (1.6–2.5)**
	High	46.0	3.8 (2.9–4.8)	3.7 (2.7–5.1)**

*Note.* *p<0.05; **p<0.01. ^†^Gender, BMI, practice type, lameness examinations, and rectal palpations had no effect in the final model.

**Table 6:** Multivariate analysis of severe MSD in the elbow.

Variables		%	Crude OR (95%CI)	Adjusted OR^†^ (95%CI)
Gender	Male	16.5	1	1
	Female	11.9	0.7 (0.6–0.8)	0.7 (0.6–0.9)*
Age (years)	<30	1.9	1	1
	30–39	6.6	3.6 (0.9–15.3)	3.8 (0.9–16.2)
	40–49	15.3	9.3 (2.3–37.9)	11.6 (2.8–47.9)**
	>50	17.2	10.7 (2.6–43.6)	13.4 (3.2–55.9)**
BMI (kg/m^2^)	<25	11.3	1	1
	25–30	17.6	1.7 (1.3–2.1)	1.3 (1.1–1.7)*
	>30	17.5	1.7 (1.2–2.4)	1.4 (0.9–1.9)
Practice type	Small	9.8	1	1
	Mixed	16.0	1.7 (1.3–2.3)	1.5 (1.1–2.1)*
	Large	19.3	2.2 (1.7–2.8)	1.9 (1.4–2.7)**
Rectal palpations	0 or n/s	10.4	1	1
	<600	11.6	1.2 (0.9–1.5)	1.0 (0.7–1.3)
	≥600	19.9	2.2 (1.7–2.9)	1.4 (1.0–2.0)*
Quantitative demands	Low	7.6	1	1
	Medium	12.5	1.7 (1.2–2.6)	1.5 (0.9–2.2)
	High	18.5	2.8 (1.8–4.2)	2.0 (1.3–3.2)**
Personal Burnout	Low	9.4	1	1
	Medium	15.4	1.8 (1.4–2.3)	1.8 (1.4–2.4)**
	High	19.1	2.3 (1.6–3.1)	2.3 (1.6–3.3)**

*Note.* *p<0.05; **p<0.01. ^†^obstetric procedures, radiography had no effect in the final model.

**Table 7:** Multivariate analysis of severe MSD in the hand/wrist.

Variables		%	Crude OR (95%CI)	Adjusted OR^†^ (95%CI)
Gender	Male	17.4	1	1
	Female	21.1	1.3 (1.1–1.5)	1.6 (1.2–2.1)**
Age (years)	<30	14.3	1	1
	30–39	16.9	1.2 (0.7–2.2)	1.1 (0.6–2.3)
	40–49	17.4	1.3 (0.7–2.2)	1.4 (0.7–2.9)
	>50	22.5	1.7 (1.0–3.1)	2.1 (1.0–4.1)*
Accidents	No	41.2	1	1
	Yes	52.0	1.5 (1.2–1.9)	1.5 (1.2–1.9)**
Rectal palpations	0 or n/s	18.0	1	1
	<600	16.6	0.9 (0.7–1.1)	0.8 (0.6–1.1)
	≥600	23.7	1.4 (1.1–1.8)	1.2 (0.9–1.7)
Quantitative demands	Low	17.3	1	1
	Medium	19.2	1.1 (0.9–1.4)	1.4 (0.9–2.1)
	High	27.5	1.8 (1.3–2.5)	1.8 (1.2–2.8)**
Personal Burnout	Low	13.0	1	1
	Medium	20.9	1.8 (1.4–2.2)	1.6 (1.2–2.1)**
	High	27.6	2.5 (1.9–3.4)	1.9 (1.4–2.9)**

*Note.* *p<0.05; **p<0.01. ^†^practice type and radiography had no effect in the final model.

Veterinarians of 40 years and older run a higher risk of having physical disability in the neck (OR 2.0, 95% CI 1.2–3.3), shoulder (OR 2.1, 95% CI 1.2–2.0), elbow (>40 years: OR 11.6, 95% CI 2.8–47.9; >50 years: 13.4, 95% CI 3.2–55.9), and hand (OR 2.1 95% CI 1.0–4.1) than their younger colleagues. Severe MSD of the hand was found more often in women (OR 1.6, 95% CI 1.2–2.1), whereas female veterinarians complained of MSD in the elbow less often than their male colleagues (OR 0.7, 95% CI 0.6–0.9). Furthermore, previous accidents increased the risk of physical impairment in neck (OR 2.1, 95% CI 1.6–2.9), shoulder (OR 1.6, 95% CI 1.2–2.0), and hand (OR 1.5, 95% CI 1.2–1.9). Veterinarians with a BMI of 25–30 had 1.3 (95% CI 1.1–1.7) times the odds of MSD severity in the elbow, compared with those who had normal BMI. Veterinarians in mixed (OR 1.5, 95% CI 1.1–2.1) and large (OR 1.9, 95% CI 1.4–2.7) practices showed a higher risk for severe MSD in the elbow than small animal practitioners.

Veterinarians who frequently performed dental procedures (≥600 per year) were at higher risk of being affected by MSD in the neck region (OR 1.4, 95% CI 1.0–1.9), compared to those who less often or hardly ever performed such tasks. Work-related risk factors for MSD in the shoulder increased significantly with the number of annual obstetric procedures (≥600 per year: OR 2.1, 95% CI 1.2–3.5). On the contrary, veterinarians who performed up to 600 radiological examinations per year less often reported severe MSD in the shoulder (OR 0.8, 95% CI 0.6–1.0). The risk of severe MSD in the elbows was associated with frequent rectal palpations (≥600 per year: OR 1.4, 95% CI 1.0–2.0). High quantitative demands and elevated levels of personal burnout showed consistent association with perceived severe MSD severity in all queried body regions, compared with those who less often reported pressure of time due to heavy workload and emotional exhaustion (Tables 4, 5, 6, 7).

## Discussion

To our knowledge, this is the first large scale self-reported survey of German veterinarians which examines MSD prevalence, its severity as manifested in restricted daily activities, and work-related accidents. In line with international literature, this study showed that MSD in the distal upper extremities and the neck was frequent in this professional group. The type of veterinary practice was related to MSD prevalence and severity. In addition, work-related accidents that resulted in MSD symptoms were most frequently reported in the hand/wrist and shoulder region. The majority of all injuries in the distal upper extremities were caused by animals. Working and individual characteristics were shown to attribute to MSD severity in the upper body.

Two studies from Australia and New Zealand using the same measuring instrument also found the highest 12-month symptoms prevalence in the neck (57%–58%) and shoulder (52%–59%), followed by the hand (32%–52%) and elbow (17%–29%) [4]. However, a Dutch veterinary cohort reported symptoms in the upper extremities significantly less often (37%, 38%, 14% and 11%, respectively) [23]. The 12-month prevalence in our study was much higher than the values found in comparable professions in international studies (e.g. nurses, farmers, physicians or chiropodists) [24]–[27]. The prevalence values are also much higher than those found in surveys with other employees. According to a survey in Germany, about 46% of subjects complained of pain in the neck and shoulder region. About 20% of employees had pain in the arms and hands [28]. In agreement with the findings of previous studies, multiple anatomical regions were often affected [1], [4], [7]. In their large-scale survey in France, Roquelaure et al. found that MSD symptoms of the upper limbs often overlapped two anatomical body regions, particularly the neck and shoulder regions [29]. The type of veterinary practice was related to MSD prevalence and severity, which is in line with the results from other studies on hazards and disorders [3], [4], [6], [8], [16], [30]. Practitioners in mixed and large animal practices much more frequently reported MSD in the upper body. In a study from the Netherlands, problems related to the musculoskeletal system of the upper body were by far the most important disorders in equine practitioners [1]. We found higher MSD prevalences for women in the neck and hand/wrist and for men in the elbow. Studies on general and working populations have reported higher MSD prevalences in woman than in men. Besides, women more often reported pain at more than one body site [31], [32].

A small but considerable number of participants had MSD symptoms which were serious enough to affect their daily activities. From the occupational perspective, this is of critical importance, as it probably affects the veterinarians’ quality of life and may also lead to changes in professional activities. This in turn may cause a loss in productivity and/or loss of earnings. The participants of the aforementioned studies less frequently reported that MSD prevented them from carrying out their daily activities than those in our study [4], [5]. To some extent, this may be attributed to the differences in the definition of items used. In this context, it should be pointed out that studies with additional diagnostic measuring procedures observed significantly lower values of MSD in the neck-shoulder region and lower back [33]–[35]. In a study on bovine practitioners, the self reported pathological findings of CTD were also lower than the prevalence of any musculoskeletal problem [6]. Thus, it would be desirable to verify the present results through further investigations with objective procedures. However, by choosing severe MSD as an outcome measure, we aimed to identify those cases with MSD complaints which were serious enough to prevent them from performing their daily activities for at least seven days in the previous 12 months.

The present study demonstrates that hand/wrist and shoulder are frequently affected by work-related accidents. A German study which analyzed the occupational records (accidents and diseases) of veterinary staff over a 5-year period found that the hand (48.3%) and arm (17.3%) were the most affected anatomical locations. They also found that veterinarians and their staff had a 2.9-fold higher risk of injuries than general practitioners [12]. In terms of species-specific injury mechanism, our results are in keeping with previous studies, which report that work-related accidents were most frequent in large animal practices and that the upper extremities were most frequently affected [3], [8], [16], [30]. Langley and Hunter analyzed data from the US Department of Labor on human workplace fatalities associated with animals for the years 1992–1997. They found that large animals (cattle and horses) were primarily responsible for the majority of fatal events among workers. Men and elderly workers were at greater risk of mortality [36]. This is similar to our findings; male and elderly veterinarians significantly more often reported work-related injuries. This is probably because male practitioners more frequently worked with large animals, which exposed them to greater risk of major injuries. However, the results with respect to gender were inconsistent. Some studies on injuries reported a greater risk for men [7], [8], while others found that women were more often affected [3], [30], [37]. Some authors have argued that women are at greater risk due to their small size and limited physical strength [3], [37]. In contrast to our results, previous findings showed that increasing years of experience were associated with decreasing injury-related events [13], [30]. This might be explained by the higher proportion of elderly veterinarians in our study. However our results on accidents were limited, because we did not differentiate the origin and severity of the accidents in detail.

Our findings demonstrate that for each part of the body a specific set of personal and work-related factors contribute significantly to severe MSD. Older age, gender, previous injuries, BMI, practice type, veterinary procedures such as dentistry, rectal palpation, obstetrics as well as quantitatively high demands and personal burnout increased the likelihood of severe MSD in the upper extremities and the neck. Older veterinarians more often reported severe MSD in all queried body parts than their younger colleagues. The age effect described in the present study on MSD is confirmed by other studies with veterinarians [4], [38]. For ageing workers, a progressive decline in physical work capacity, characterized by diminished muscular capacity, has been reported – especially for physically demanding occupations [39]. In general, the MSD are likely to become more prevalent in the veterinary profession as the working population ages and a shortage of young professionals is to be expected, especially in the large animal practices [40]. With respect to gender, women showed a higher risk of severe MSD in the hand and men in the elbow. This could be due to different mechanical patterns of procedures undertaken and/or differences in physique. According to Berry et al., women and men reported different procedures which caused them CTD; men more often reported rectal palpation and calving manipulation while women reported holding instruments, computer work and other causes [3]. The activity profile that causes MSD is greatly dependent on the type of practice. Scuffham et al. asked veterinarians about routine activities that triggered MSD in them. Large and mixed animal veterinarians mostly considered that rectal examinations, obstetric treatment, ultrasound examinations and diagnostic testing on the hoof and lameness were stressful activities. On the other hand, small animal veterinarians found that lifting and transporting animals was stressful, together with surgeries [41]. In a subsequent study, it was shown that large animal veterinarians and veterinarians who only worked with horses exhibit the greatest prevalence of MSD periods in comparison to veterinarians in other practices or organizations [4]. Cattell found that 71% of CTD and 31% of ATI to veterinarians in large animal practices were related to rectal examinations [6]. Thus, in view of the activity profile, it is plausible in the present study that mixed and large animal veterinarians, who also performed rectal palpations, significantly more often reported symptoms in the elbows. Symptoms in the shoulders correlate with frequent vaginal investigations, whereby the type of practice played a lesser role in the corresponding analysis model. In addition, dental examinations correlated with symptoms in the neck. It is known that this region is susceptible for MSD - not only for human dentists, but also for veterinarians specializing in dentistry [42], [43].

Furthermore, participants in our study who reported previous work-related accidents had a higher chance of developing MSD in the neck, shoulder and hand/wrist. These results were also supported by Randall et al., who examined ergonomic risk factors among veterinary sonographers [38]. Thus, trauma acquired at work may have serious long-term consequences. Precautionary measures, such as training in body posture and handling animals, are of increasing importance in this profession. Ergonomics of the work environment can also be considered to decrease the incidence of injuries to the neck (e.g. for practitioners that have higher incidence of neck pathology).

In the current study, quantitative demands and personal burnout were associated with significant increases in MSD in all queried body regions. The association between psychosocial factors and MSD is well documented [44]. The relationship between MSD period prevalence and quantitative demands caused by time pressure and work overload was consistent with two recent studies from Australia and New Zealand [4], [5]. The assumption is that a mismatch of the amount of work and the time available to do it may lead to stress [45]. Thus, MSD is not only triggered by physical factors (e.g. lifting, repetitive tasks), but also by emotional and psychosocial demands. For instance, Loomans et al. found an intermediate correlation in veterinarians between emotional work load and MSD of the lower body [1]. In addition, psychosocial stress in the veterinary setting is also associated with increased consumption of alcohol, tobacco and medication [46]. For these reasons, it is strongly recommended that preventive measures should be implemented to sustain and improve not only the physical but also the psychological well-being of veterinarians. However, we cannot rule out the possibility of reverse causation. Although the cross-sectional design is sufficient for making initial associations, it is ineligible to derive causal relationships.

### Strengths and Limitations

Our study includes a large number of participants and is one of the most extensive international studies of veterinarians. The response rate of 38.4% corresponds well with the average response rate of 38.5% in other studies with veterinarians [4]. By using registration data, the sample size can be considered representative with respect to gender and age. The use of a common standardized questionnaire enables us to compare these results with other studies among veterinarians and other occupational groups. However, some limitations should be pointed out. The retrospective data collection of exposure and complaints is susceptible to recall bias, because of the potential for misreporting the number of veterinary procedures and MSD related events in the preceding 12 months. Our study, like others, was limited by the inability to survey the non-respondents in depth due to methodological and organizational issues. We cannot rule out the possibility that veterinarians who suffered from MSD had greater motivation to participate in the study than those who were not affected, so that we were prone to overestimate the MSD burden in this occupational group. In addition, a healthy worker effect might potentially have resulted in minor underestimation of MSD, as some veterinarians might previously have left their occupation or remained on sick leave due to MSD or other diseases. The results may have been influenced by other potential work-related factors which were not considered when collecting data, in particular, insufficient recreation time, career structure, client interaction, perceived peer support, use of auxiliary devices or specific work activities (e.g. working in cold environments or working postures) [4], [5]. This lack of data limits the ability to broadly explore the association between work-related factors and severe MSD in the upper extremities and the neck. A further limitation consists in the redundant predictors. As some veterinary procedures correlated highly with each other, we omitted a few explanatory variables in the multivariate models. The omission of the variables may limit the explanatory power of the model. However, we did not observe significant changes in the R-squared values of the analyzed models when we removed these factors. In general, causal relationships between variables cannot be derived in cross-sectional studies, although these allow us to quantify the magnitude of MSD prevalence and to identify initial associations. In order to establish causal explanations for MSD, a longitudinal study will be required, which has not yet been performed in this occupational group.

## Conclusions

Our study contributes to the available evidence on the MSD and shows that these disorders are highly prevalent in the upper body of German veterinarians. The overall prevalence appears to be similar to that found in other international studies. From the perspective of occupational health and safety, it seems to be necessary to improve accident prevention and to optimize the ergonomics of specific tasks. In order to prevent MSD in the upper body, our data suggest the need for target group-specific (e.g. gender and practice type) preventive measures that also focus on the psychological factors at work. As a consequence of demographic changes, employees are remaining in their professions for longer. In particular, this applies to independent veterinarians in Germany, which emphasized the importance of preventive measures. Veterinary associations and organizations should provide their members with adequate training and information, so that they can work safely with animals and equipment. Preventive measures should be incorporated into the curriculum and explain to future veterinarians about the permanent and seasonal challenges in the individual veterinary practices and the related activities. Further research work must concentrate on the long-term consequences of veterinary work for the musculoskeletal system. The resulting knowledge could, for example, provide evidence-based aids for decision when determining and evaluating exposure in occupational diseases.
